# Postpartum Stroke: The Importance of Early Recognition and Multidisciplinary Management

**DOI:** 10.7759/cureus.116751

**Published:** 2026-09-22

**Authors:** Falak Naz Baloch, Maryam Javed, Nida Khalid, Nicola Roberts, Liliana Grosu

**Affiliations:** 1 Department of Obstetrics and Gynaecology, Bedfordshire Hospitals NHS Foundation Trust, Bedford, GBR; 2 Department of Obstetrics and Gynaecology, Hameed Latif Hospital, Lahore, PAK

**Keywords:** acute ischaemic stroke, caesarean section, maternal stroke, postpartum stroke, pregnancy-associated stroke

## Abstract

Pregnancy and the postpartum period are associated with an increased risk of stroke. We report the case of a 41-year-old para 6 woman with pre-existing hypertension who underwent a category 2 caesarean section at term for fetal distress. A few hours postoperatively, a family member noticed that she was more drowsy than usual. Subsequent clinical assessment revealed left-sided limb weakness and facial drooping. Prompt clinical assessment and multidisciplinary escalation were initiated, followed by transfer to a specialist stroke unit within the potential therapeutic window for reperfusion therapy. Computed tomography (CT) scan of the head and CT angiography showed no obvious thrombus or large-vessel occlusion. Magnetic resonance imaging (MRI) confirmed an acute ischaemic infarction involving the right basal ganglia and subcortical right frontal lobe. Magnetic resonance venography (MRV) showed no evidence of cerebral venous sinus thrombosis (CVT). Bilateral carotid Doppler ultrasonography was normal. Intravenous thrombolysis was considered by the stroke team but was not recommended, while mechanical thrombectomy was not indicated because there was no large-vessel occlusion. This case highlights the importance of maintaining a high index of suspicion for acute neurological deterioration in the immediate postpartum period, particularly in women with underlying vascular risk factors. Early recognition, prompt multidisciplinary escalation, and timely specialist management are essential to optimise outcomes in postpartum patients with suspected stroke.

## Introduction

Pregnancy and the postpartum period are associated with an increased risk of stroke [1]. Stroke is the fourth leading cause of death among women overall and the eighth leading cause of pregnancy-associated mortality [2]. Pregnancy-associated stroke has been reported to occur at an estimated rate of 34.2 per 100,000 births, with evidence suggesting an increasing incidence [2]. Women with hypertensive disorders of pregnancy have been reported to have more than five times the risk of stroke compared with those without hypertensive disorders [3]. Furthermore, pre-existing hypertension is associated with an increased risk of pre-eclampsia, eclampsia, and stroke during pregnancy [4].

Pregnancy induces physiological changes that increase the risk of stroke, including a hypercoagulable state, haemodynamic alterations, and changes in vascular function. These adaptations, together with factors such as venous stasis and the prothrombotic state associated with the peripartum period, may increase the risk of thromboembolic events, including ischaemic stroke [5]. Optimisation of modifiable cerebrovascular risk factors, particularly hypertension, is important during pregnancy to reduce the risk of stroke and hypertensive complications [6].

We report the case of a 41-year-old para 6 woman with pre-existing hypertension who developed acute neurological deterioration in the immediate postpartum period following a caesarean section for fetal distress. She was promptly assessed with multidisciplinary escalation, with subsequent neuroimaging confirming an acute ischaemic infarction involving the right basal ganglia and subcortical right frontal lobe.

## Case presentation

A 41-year-old para 6 woman, with all previous deliveries by spontaneous vaginal delivery, was admitted at term. Her pregnancy was complicated by pre-existing hypertension, for which she was taking nifedipine 20 mg twice daily. Her booking BMI was 32 kg/m². Her past medical history was also significant for asthma. Anti-M antibodies were detected during pregnancy. Her blood group was O positive.

She underwent a Category 2 caesarean section under spinal anaesthesia at term for fetal distress. The estimated intraoperative blood loss was 675 mL. She required atropine for bradycardia during the perioperative period, with no documented hypotension. Following surgery, the neonate was admitted to the neonatal unit, and the patient was transferred to the postnatal ward following an initial period in recovery.

Approximately five hours after the caesarean section, a family member reported that the patient appeared more drowsy than usual. The attending doctor was contacted and promptly attended the postnatal ward to assess the patient. Clinical assessment identified drowsiness associated with new left-sided limb weakness and left facial drooping, representing a change from her expected postoperative condition and raising suspicion of an acute cerebrovascular event. An A-E assessment (Airway, Breathing, Circulation, Disability, Exposure) and neurological examination were performed, and the obstetric, anaesthetic, medical and stroke teams were contacted promptly for further assessment and management.

Given the time-critical nature of suspected acute stroke, the patient was transferred to the specialist stroke unit at a tertiary care hospital for urgent assessment and consideration of reperfusion therapy. CT of the head and CT angiography were performed following transfer. CT angiography demonstrated no obvious thrombus or large-vessel occlusion; therefore, mechanical thrombectomy was not indicated. Intravenous thrombolysis was considered by the stroke team but was not recommended. The specific clinical rationale for this decision was not documented in the available case records. Further assessment with MRI and MR venography was subsequently planned.

Table 1 lists the timeline of key clinical events.

**Table 1:** Timeline of key clinical events from caesarean section to diagnosis and management of acute ischaemic stroke

Date/time	Clinical event
Day 0	Category 2 caesarean section performed under spinal anaesthesia for fetal distress
Approximately 5 hours later	Family member noticed increased drowsiness
Shortly thereafter	Medical team contacted; urgent bedside assessment initiated
Subsequently	Left-sided limb weakness and facial drooping identified; multidisciplinary escalation to obstetric, anaesthetic, medical and stroke teams
Subsequently	Transferred to specialist stroke unit for urgent assessment and consideration of reperfusion therapy
Later that day	CT head and CT angiography performed; no obvious thrombus or large-vessel occlusion identified
Day 3	MRI/MRV demonstrated acute ischaemic infarction involving the right basal ganglia and subcortical right frontal lobe; no cerebral venous sinus thrombosis
Day 4	Bilateral carotid Doppler showed no haemodynamically significant stenosis
Day 8	Discharged following clinical improvement, with stroke clinic follow-up arranged

MRI of the brain performed on day 3 demonstrated areas of acute ischaemic infarction involving the right basal ganglia and subcortical right frontal lobe. MR venography showed no evidence of cerebral venous sinus thrombosis. Blood glucose was 4.8 mmol/L, and bilateral carotid artery Doppler ultrasonography showed no haemodynamically significant stenosis in either extracranial carotid artery. A thrombophilia and autoimmune workup was also performed. Lupus anticoagulant was not detected, while anticardiolipin IgG and IgM antibodies, β2-glycoprotein I IgG and IgM antibodies, antineutrophil cytoplasmic antibodies (ANCA), including myeloperoxidase and proteinase 3 antibodies, and sickle cell solubility testing were negative. Platelet counts remained within the normal range. The relevant investigations are summarised in Table 2.

**Table 2:** Summary of neuroimaging, vascular, haematological, thrombophilia and autoimmune investigations performed during evaluation of acute ischaemic stroke ANCA: antineutrophil cytoplasmic antibodies

Investigation	Result
CT head and CT angiography	No obvious thrombus or large-vessel occlusion identified
MRI brain	Acute ischaemic infarction involving the right basal ganglia and subcortical right frontal lobe
MR venography	No evidence of cerebral venous sinus thrombosis
Bilateral carotid Doppler	No haemodynamically significant stenosis
Lupus anticoagulant	Not detected
Anticardiolipin IgG/IgM	Negative
β2-glycoprotein I IgG/IgM antibodies	Negative
ANCA (MPO/PR3)	Negative
Sickle cell solubility test	Negative
Platelets	Normal throughout admission
Blood glucose	4.8 mmol/L
Prothrombin time	11.6 seconds

Following the diagnosis of acute ischaemic stroke, the patient was commenced on aspirin for secondary prevention and remained under the care of the stroke team. She showed significant clinical improvement and was discharged on day 8 following the caesarean section, with follow-up arranged in the stroke clinic.

## Discussion

This case highlights the diagnostic challenge of recognising acute ischaemic stroke in the immediate postoperative and postpartum period, when neurological deterioration may initially be attributed to postoperative recovery, anaesthesia, or other peripartum factors. The educational value of this case lies in emphasising the importance of maintaining clinical suspicion when new neurological symptoms occur after delivery, particularly in women with established vascular risk factors. Prompt neurological assessment and multidisciplinary escalation are essential to facilitate timely neuroimaging and assessment for reperfusion therapy. In this patient, early recognition of the neurological deterioration led to urgent specialist stroke assessment and neuroimaging, allowing reperfusion treatment options to be considered without avoidable delay.

Although stroke during pregnancy and the postpartum period is uncommon, it can have devastating consequences, resulting in significant maternal morbidity and mortality as well as adverse fetal outcomes [7]. The early postpartum period is associated with an increased risk of thrombotic events, with the risk remaining elevated during the first six weeks following delivery [8].

Advanced maternal age, hypertension and obesity are recognised cerebrovascular risk factors in pregnancy, highlighting the importance of early identification and optimisation of modifiable risk factors [9,10]. In our patient, the coexistence of these risk factors may have contributed to her overall cerebrovascular risk, although a direct causal relationship cannot be established from a single case.

Recognition of stroke in the peripartum period can be challenging because presentations may be variable and include non-specific symptoms. Symptoms such as confusion, headache and altered consciousness may overlap with other pregnancy-related conditions, potentially contributing to missed or delayed diagnoses. Recent evidence suggests that missed diagnostic opportunities occur in a substantial proportion of pregnancy-associated stroke cases, with failure to recognise symptoms and omission of appropriate neuroimaging identified as important contributing factors [11]. Failure to recognise stroke promptly can delay time-sensitive treatment and may adversely affect clinical outcomes. 

In a review of pre-eclampsia-related maternal deaths in California, delayed response to clinical warning signs was identified as a potential contributor to poor outcomes in 91% of stroke-related deaths, highlighting the importance of prompt recognition and management of neurological symptoms [12]. Although this cohort specifically examined pre-eclampsia-related maternal deaths, its findings reinforce the potential consequences of delayed recognition of neurological deterioration in the peripartum period.

Acute ischaemic stroke is a time-critical neurological emergency, and pregnancy or the postpartum state should not delay appropriate evaluation for reperfusion therapy. Eligible patients should be assessed promptly for intravenous thrombolysis and/or mechanical thrombectomy according to standard stroke pathways [5]. In our patient, prompt clinical assessment and multidisciplinary escalation involving the obstetric, anaesthetic, medical, and stroke teams facilitated transfer to a specialist stroke unit for urgent stroke assessment and consideration of reperfusion therapy. Intravenous thrombolysis was considered by the stroke team but was not recommended, while mechanical thrombectomy was not indicated because CT angiography showed no large-vessel occlusion.

This case also highlights the importance of effective communication and continuity of handover during time-critical clinical deterioration. During the initial assessment, the midwife who had identified the patient's deterioration was unavailable, while the midwife accompanying the assessment had not received the relevant clinical handover from recovery. Although this resulted in only a short delay in obtaining additional clinical information, it illustrates how gaps in communication can potentially affect the efficiency of assessment and decision-making during a time-sensitive emergency.

Neuroimaging confirmed acute ischaemic infarction involving the right basal ganglia and subcortical right frontal lobe, correlating with the patient's left-sided neurological deficits. Bilateral carotid Doppler ultrasonography was normal, with no evidence of significant carotid disease. CT angiography also demonstrated no evidence of thrombus or large-vessel occlusion, while MR venography showed no evidence of cerebral venous sinus thrombosis. Although a definitive underlying mechanism for the infarction was not identified, the patient's pre-existing hypertension, obesity, advanced maternal age and the physiological prothrombotic state of the postpartum period may have contributed to her overall cerebrovascular risk.

The negative thrombophilia and autoimmune investigations, including antiphospholipid antibodies, lupus anticoagulant and ANCA testing, did not identify an alternative underlying cause. However, the absence of an identified mechanism does not exclude other potential contributors to the stroke.

## Conclusions

Acute ischaemic stroke is an uncommon but potentially serious complication of pregnancy and the postpartum period. This case highlights the diagnostic challenge of recognising stroke in the immediate postoperative period and the importance of prompt neurological assessment, multidisciplinary escalation and timely specialist stroke evaluation to preserve access to reperfusion therapy. Particular vigilance is warranted in postpartum women with established vascular risk factors.
